# Hemorrhoidal disease: what role can rectal artery embolization play?

**DOI:** 10.3389/fsurg.2024.1474799

**Published:** 2025-01-07

**Authors:** Julien Panneau, Diane Mege, Mathieu Di Bisceglie, Julie Duclos, Idir Khati, Vincent Vidal, Gaetano Gallo, Farouk Tradi

**Affiliations:** ^1^ Interventional Radiology Section, Department of Medical Imaging, University Hospital Timone, APHM, Marseille, France; ^2^ Aix Marseille Univ, LIIE, Marseille, France; ^3^ Aix Marseille Univ, CERIMED, Marseille, France; ^4^ Department of Visceral Surgery, Aix-Marseille University, Hôpital de la Timone, Marseille, France; ^5^ Department of Surgery, Sapienza University of Rome, Rome, Italy

**Keywords:** hemorrhoids, embolization, emborrhoid, hemorrhage, radiology, interventional

## Abstract

**Introduction:**

Hemorrhoidal artery embolization, also known as Emborrhoid, has emerged in recent years as a minimally invasive treatment option for patients with recurrent and unresponsive to medical therapies hemorrhoidal bleeding symptoms. We present here an overview of the profile of rectal artery embolization based on the most relevant and recent literature.

**Methods:**

A comprehensive review of literature on Hemorrhoidal artery embolization, was conducted on PubMed–Medline. The most relevant literature was summarized narratively.

**Results:**

Current literature confirms the feasibility, efficacy and safety of rectal artery embolization for bleeding hemorrhoids. To date, the results of nearly 250 patients who have undergone hemorrhoid embolization have been published in several studies. All these authors have reported high immediate technical success, with also high clinical success, ranging from 63% to 94%, without major complications. Because of its beneficial safety profile, rectal artery embolization represents an attractive option for selected patients. In case of recurrence of bleeding it is possible to consider repeating the embolization procedure. Treatment failure may be due to the presence of anatomical variants such as dominant middle rectal arteries, which can be investigated and treated in the second session if necessary.

**Conclusion:**

Rectal artery embolization represents a valuable addition to the therapeutic armamentarium of bleeding hemorrhoidal disease, if patients are selected appropriately.

## 1 Background

The technique of rectal artery embolization has emerged as a new treatment option over the past decade for patients suffering from bleeding hemorrhoids. Hemorrhoidal disease is the most common anorectal disorder, affecting millions of people worldwide, with a prevalence ranging from 4.4% to 39% (1, 2) and predominant symptoms of bleeding and pain (3–5). The internal hemorrhoids are a physiological vascular structure composed of a richly anastomosing arteriovenous network that contributes to the continence of the anal canal, forming a circumferential submucosal bulge at the anorectal junction, known as the corpus cavernosum recti (CCR) (6–9). Although the pathophysiology remains controversial and multifactorial, internal hemorrhoidal pathology is thought to result from chronic hypertrophy of these vascular structures secondary to a chronic increase in flow through the CCR, ultimately leading to hyperplasia of the cushions responsible for congestive hypertension which can be considered as one of the main factor (10, 11).

Most patients are relieved by medical or minimally invasive treatments (injection sclerotherapy, rubber band ligation, infrared photocoagulation, bipolar diathermy), and surgical treatment is required in 10% of cases (12, 13). The gold-standard surgical procedure is the open hemorrhoidectomy of Milligan and Morgan (14–16). Today, however, there are less invasive techniques such as the Longo procedure. The hemorrhoidopexy with or without doppler-guided elective hemorrhoidal artery ligation (DG-HAL) (17–21) always in the context of a tailored approach. Following the principle of less invasivity emborrhoid introduced by vidal et al. in 2013 (22), is gaining a strong role in the surgical option panorama.

Here we discuss rectal artery embolization, its advantages and disadvantages, based on the most recent and relevant literature, and attempt to clarify its role in the treatment of hemorrhoidal disease.

## 2 Patient selection

Selecting and monitoring patients who will benefit from an embolization procedure requires a multidisciplinary team of interventional radiologists and proctologists. An initial preoperative clinical examination by a proctologist or visceral surgeon is essential to assess the stage of hemorrhoidal disease and to verify the absence of anorectal cancer. At the initial consultation, the severity of hemorrhoidal symptoms is assessed using the Hemorrhoidal Bleeding Score (23), which ranges from 0, indicating that the severity of hemorrhoidal symptoms has not been assessed, to 9, indicating daily bleeding with anemia, requiring a blood transfusion (Table 1). The degree of internal prolapse is assessed using the Goligher classification (24, 25), ranging from 1 (no prolapse) to IV (irreducible prolapse) (Table 2). It is necessary to describe the number of pathological piles and the clinical characteristics of each of them. In particular the degree of Goligher but also which piles bleed and which ones do not bleed (20, 21).

**Table 1:** Hemorrhoidal bleeding score.

Variable	Score
Frequency	
Never	0
<1/day or at each bowel movement	1
≥1/day or at each bowel movement	2
Type	
Never	0
Wiping ± underwear	1
Toilet bowl	2
Anaemia	
Never	0
Iron deficiency without anemia	1
Without transfusion	2
With transfusion	3
Discomfort	
Little or no discomfort	0
Moderate discomfort	1
Extreme or permanent discomfort	2
Overall Score	_

**Table 2:** Goligher's classification.

Grade	Description
Grade I	Hemorrhoids without prolapse
Grade II	Prolapse on defaecation with spontaneous reduction
Grade III	Prolapse on defaecation requiring manual reduction
Grade IV	Irreducible hemorrhoids

The best candidates for rectal artery embolization are those with grade II-III prolapse and whose predominant symptoms are hemorrhagic in nature, as well as patients with grade IV prolapse in cases of surgical contraindication.

It is important to take a detailed history of the patient, which should include the extent, severity and duration of symptoms, daily eating habits, associated symptoms (e.g., fecal incontinence, constipation) and details of bowel movements. Rectal artery embolization is indicated for patients suffering from recurrent and unresponsive hemorrhoidal bleeding. This may be of interest in the case of patients on long-term anticoagulant or antiplatelet therapy, as well as patients with coagulation disorders (e.g., hemophilia). Chronic inflammatory bowel disease, on the other hand, is not a good indication, as these patients essentially suffer from anal fistulas or digestive tract lesions unrelated to hemorrhoidal lesions. However, embolization of the rectal arteries will not alleviate the normal ageing process or any positional pelvic disorders.

The preprocedural evaluation should rule out the presence of conditions that are general contraindications to hemorrhoid embolization, such as anorectal cancer, as well as those that are contraindications to conventional angiography, like renal failure and allergies to iodinated contrast media. Advanced atherosclerosis is a relative contraindication that could result in technical failure and should be assessed during preprocedural imaging.

## 3 Procedure

The patient is given detailed explanations about the procedure, its benefits, and risks. Written consent is obtained. The patient may receive light sedatives to reduce anxiety if it's necessary. No prophylactic antibiotics required. The puncture site (usually the groin area for access to the femoral artery) is shaved and disinfected.

**Figure 1:**
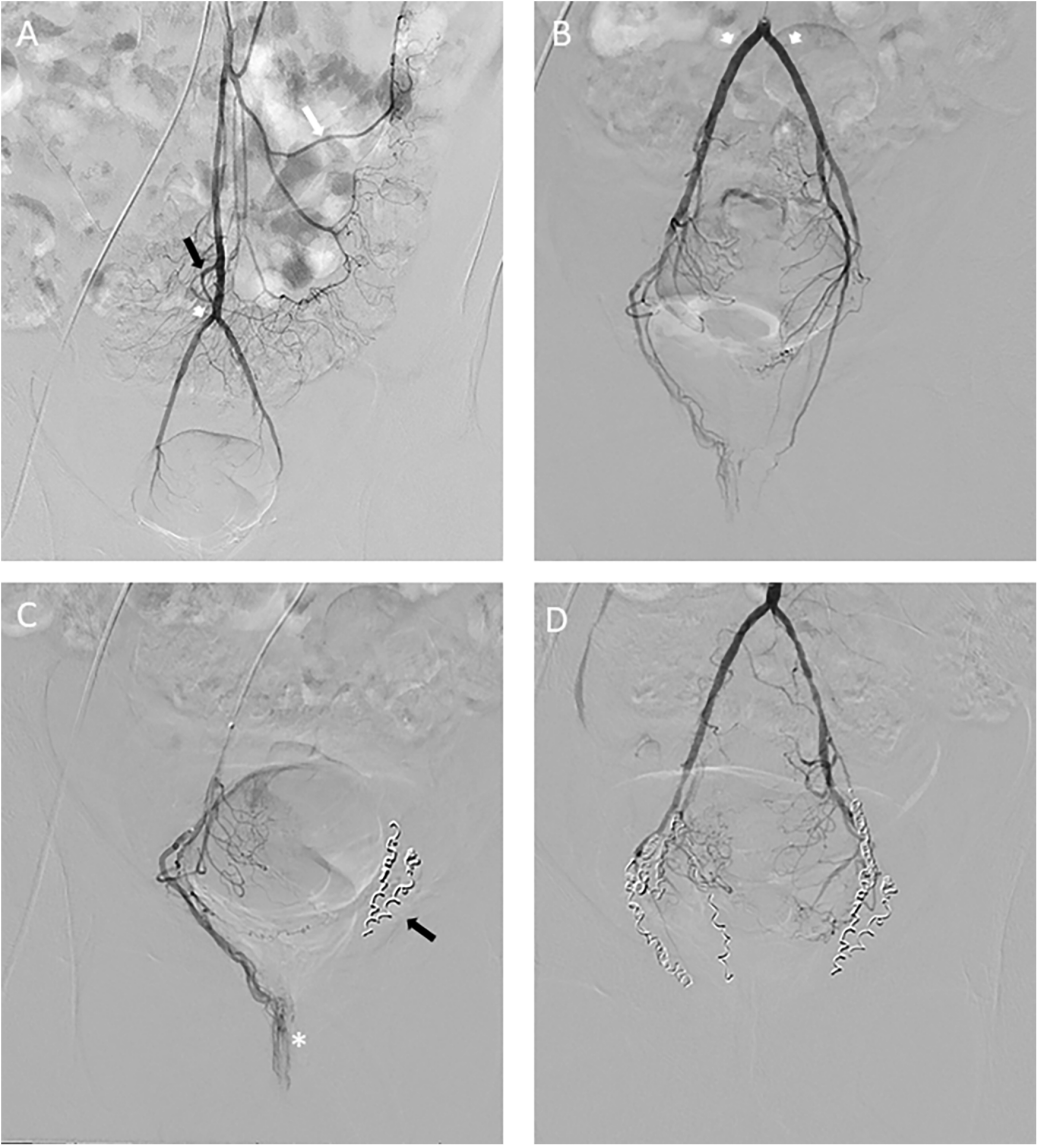
**(A)** Frontal digital subtraction angiography showing a modal anatomy of the inferior mesenteric artery, in order, the left colonic artery (white arrows) and then the sigmoid artery (black arrows). Then comes the SRA (arrowheads) terminal branch of the IMA that ends in projection from the pubic bone. **(B)** Frontal DSA showing codominant right and left SRA anterior branches (arrowheads). **(C)** Progression of the microcatheter to the tortuous right anterior branch, projecting opposite the pubic bone (asterisk) and embolization of the left SRA artery (black arrow) **(D)** At the end of the procedure, no residual branch is opacified under the pubic symphysis.

Under local anaesthetic, the femoral artery is punctured and a valved introducer inserted. Transradial access can also be used, and can provide greater patient satisfaction and fewer complications than transfemoral access (26, 27). The radiologist uses real-time imaging (fluoroscopy) and cone-beam CT to guide the catheter through the arterial system to the inferior mesenteric artery and finally to the superior rectal arteries (28). Once the catheter is in place, a contrast agent is injected to visualize the rectal arteries and identify the branches supplying the hemorrhoids. The radiologist specifically targets the branches of the superior rectal arteries that supply the hemorrhoids. Microbeads or metal coils are injected through the catheter to occlude these arterial branches, thereby reducing blood flow to the hemorrhoids (29) (Figure 1).

After embolization, the catheter is carefully removed. Pressure is applied to the insertion site to stop any bleeding. Sometimes, a vascular closure device is used to seal the incision (30). A compression dressing. The patient is monitored for a few hours to observe any signs of immediate complications, such as bleeding or an allergic reaction to the contrast agent (31). Instructions are provided on caring for the insertion site, managing pain, and recognizing signs of potential complications.

The patient can return home the same day after the interventional radiologist's visit if except in cases where the patient prefers to stay overnight in hospital under supervision.

## 4 Efficacy

Studies show that rectal artery embolization can significantly reduce symptoms. The technical success rate of rectal artery embolization is high, indicating that radiologists can usually successfully occlude the targeted arteries without major complications. Patients treated with rectal artery embolization often report high levels of satisfaction due to the minimally invasive nature of the procedure and rapid symptom relief (32–40).

Makris et al. provide an extensive review of multiple studies assessing the efficacy and outcomes of rectal artery embolization in treating hemorrhoidal disease (41). Fourteen studies involving a total of 362 patients were analyzed. The mean maximum follow-up duration was 12.1 months, and the average hospital stay was 1.5 days. The mean technical success rate was 97.8% (SD 3.5). A significant reduction in the French bleeding score was observed before and after embolization (*P* = 0.004). The effect on pain was reported in 6 of 14 studies, and all of them reported significant relief; however, this finding was quantified with various questionnaires. Bleeding recurrence during the follow-up period was reported in 11 of the 14 studies, with an average occurrence in 22.5% of patients (range, 5.4%–44%). Among this subgroup, 5%–43% of patients required embolization of additional vessels, such as the inferior rectal artery (IRA) or middle rectal artery (MRA). No cases of bowel ischemia, necrosis, or anorectal complications were reported.

Another meta-analysis published by Nguyenhuy looked at 14 different studies involving some 400 patients (42). This study confirms a success rate of rectal embolization between 73% and 89% with no major complication.

Compared to traditional surgical treatments such as hemorrhoidectomy, rectal artery embolization offers several advantages (even if a comparison with transanal desarterialization without pexia or sclerotherapy seems more appropriate):

- Less Postoperative Pain: Patients generally experience less pain after embolization compared to hemorrhoidectomy.
- Quicker Recovery: The procedure is less invasive, allowing for a quicker return to normal activities.
- Fewer Complications: The complication rate is generally lower compared to traditional surgical interventions.

## 5 Complications and follow-up

Rectal artery embolization is generally accepted to have a very beneficial side effect profile. Complications at the puncture site, such as hematoma, infection, and pseudoaneurysm, may occur but are rare, particularly when using ultrasound-guided vascular access and a closure device. There have been no reported anal complications associated with nonterminal occlusion using microcoils, and no cases of sphincteric dysfunction have been reported to date.

Anal discomfort, such as tenesmus, may occur during the procedure but typically resolves spontaneously. It is crucial to exercise caution when using microspheres in hemorrhoid embolization to prevent any ischemic complications while treating a benign condition like hemorrhoids.

After embolization, each patient is monitored by the interventional radiologist, proctologist, or surgeon to evaluate changes in symptoms using the hemorrhoidal bleeding score, Goligher classification, and a quality of life score. Any complications approximately three months after the procedure are recorded using the Clavien–Dindo grading system (grades I–IV) for surgical complications (43, 44). During the 3-month follow-up visit, patients are reassessed using the 36-item short-form health survey and the hemorrhoidal bleeding score. If there are improvements and the patient is satisfied, the procedure is deemed successful. In cases of procedural failure, a new embolization may be proposed to identify any residual artery.

## 6 Conclusion

Rectal artery embolization is a minimally invasive treatment option for patients with bleeding hemorrhoidal disease. Safety and efficacy have been adequately proven in the short and medium term. Because its therapeutic approach is completely different from other techniques, rectal artery embolization also has some unique features.

Hemorrhoidal artery embolization should target patients with recurrent bleeding as their main symptom. This technique has some limitations, as it does not improve the prolapse itself, and should only be used in cases where painful symptoms are at the forefront, when all other pathologies have been eliminated and usual treatments ineffective. Collaboration with proctologists and visceral surgeons is crucial. The aim is not to replace existing surgical therapies but to provide an additional treatment option for patients.

The most important advantages are the absence of post-operative complications, particularly post-operative pain, the ambulatory nature of the management and the high efficacy. If patients are selected appropriately, hemorrhoidal artery embolization represents a step towards more patient-oriented treatment, and a valuable addition to the therapeutic arsenal in the treatment of chronic hemorrhoidal disease. Randomized controlled trials with longer follow-up are needed to determine the optimal role of this emerging and minimally invasive technique. In addition, the place of different embolization materials needs to be assessed in a comparative study. The latest studies show superior clinical success rates when using microparticles, with lower re-intervention rates compared with coils.
